# How Job Creativity Requirements Affects Employee Creativity: Evidence From a Across-Level Analysis

**DOI:** 10.3389/fpsyg.2021.714886

**Published:** 2021-12-28

**Authors:** Yingshuang Ma, Haomin Zhang, Yi Dai

**Affiliations:** ^1^Institute of Business, Huanggang Normal University, Huanggang, China; ^2^School of Management, Shanghai University, Shanghai, China; ^3^Business School, Shanghai Dianji University, Shanghai, China

**Keywords:** job creativity requirement, creative process engagement, employee creativity, team knowledge sharing, Pygmalion

## Abstract

The present study adopted the Pygmalion perspective and a multilevel theoretical framework to investigate whether creative process engagement mediates the linkage of job creativity requirement with employee creativity. We examined whether team knowledge sharing moderates the aforementioned relationship. We obtained data from 71 supervisors and their 453 employees from three companies in China and applied Hierarchical Linear Modeling (HLM) version 6.08 to test the cross-level hypotheses. The results revealed that creative process engagement mediates the positive linkage of job creativity requirement with employee creativity. In addition, we observed that team knowledge sharing moderates the relationship among job creativity requirement, employee creativity, and creative process engagement. The practical and theoretical implications of the findings are discussed.

## Introduction

As competition among enterprises becomes more and more intense, creative problem solving becomes more and more important in work. Organizations expect employees to solve problems creatively. Why can creativity be stimulated? Is it like saying you can, then you can? Creativity refers to the generation of innovative and useful ideas (Zhou and George, 2001). In the global market, creativity is vital for innovation, efficacy, and survival (Jiang and Gu, 2017; Kim and Choi, 2017). Studies should identify factors that enhance employees’ creativity (Unsworth and Clegg, 2010; Zhou and Hoever, 2014; Hughes et al., 2018; Kwan et al., 2018). Creative performance can fulfill the demand of creativity requirement in a job. The demand of creativity requirement from team, namely, job creativity requirement, is specifically defined as part of the job description that encourages task complexity, autonomy, and creativity (Unsworth et al., 2005). Considering that job creativity requirement can predict employees’ creativity (Yuan and Woodman, 2010; Shin et al., 2017), only a few studies has investigated how job creativity requirement affects employees’ creativity (Tierney and Farmer, 2011; Anderson et al., 2014).

Job creativity requirement is usually the part of a job description (Unsworth et al., 2005). Job requirements can enhance the innovative behavior of employees. Researchers have explored the antecedents and outcomes of job creativity requirement considering its importance. For example, a study argued that work factors, namely autonomy, leader and innovation support, and time demands, serve as the antecedents of creative requirement (Unsworth et al., 2005). Furthermore, Hon (2013) suggested that job creativity requirement can propel employees to enhance their service performance owing to job stress resulting from the particular requirement. Although vital insights into job creativity requirement and employee creativity have been provided by studies, mechanisms underlying the effect of job creativity requirement on employee creativity remain elusive (Shin et al., 2017). Unsworth et al. (2005) elucidated mechanisms through which job creativity requirement affects employee creativity. However, they did not evaluate boundary conditions under which job creativity requirement affects employee creativity. Although a study (Shin et al., 2017) examined boundary conditions under which employee creativity is increased by job creativity requirement, they did not elucidate mechanisms through which job creativity requirement affects employee creativity.

The aforementioned studies did not provide a theoretical framework that explains processes and conditions through which job creativity requirement promotes employee creativity. Furthermore, limited research on employee creativity and job creativity requirement has been conducted in non-Western cultures (e.g., Chinese culture). Therefore, in the present study, we proposed a framework that explained the association of job creativity requirement with employee creativity and provided information regarding some crucial intervening variables.

The proposed framework elucidated mechanisms through which the requirement of job creativity promotes the creativity of employees. Amabile et al. (1996) indicated that participating in creative activities can considerably effect employee creativity behavior. Participating in creative activities, namely, creative process engagement, which represents a necessary first step toward creativity (Gilson and Shalley, 2004). Hence, creative process engagement may mediate the relationship between job creativity requirement and employee creativity. We developed this model by reviewing the creativity literature and adopted Pygmalion theory to propose that creative process engagement mediates the association of job creativity requirement with employee creativity. Furthermore, another study (Gilson et al., 2013) reported individuals’ development and creativity are constantly molded through knowledge exchange within a team. Knowledge sharing may help individuals improve their creative potential (Dong et al., 2017). Therefore, we proposed that knowledge sharing within a team can conditionally moderate the association between employee creativity and creative process engagement. In addition, we determined how job creativity requirement affects employee creativity through engagement in the creative process.

This study contributes to the existing literature in three aspects. First, we extend previous creativity research by identifying a different mechanism (i.e., Pygmalion mechanism) linking job creativity requirement and employee creativity. Specifically, we suggest that job creativity requirement will influence employee creativity by triggering a Pygmalion process that strengthens intrinsic motivation to work creatively. Our study theorizes and reveals the conditions under which perceived job creativity requirement is interpreted as desirable via the Pygmalion process and hence positively related to employee creativity. Our study thus responds to the call for more research on the mechanisms by innovation requirements affect employee creativity (Jiang and Gu, 2017; Shin et al., 2017). Second, our study contributes to both the job creativity requirement and the creativity literatures by examining and confirming creative process engagement as a mediating mechanism through which job creativity requirement ultimately influences employee creativity. We show how job creativity requirement translate into employee creativity through the Pygmalion process, our research addresses the need for research into the internal mechanisms that link job creativity requirement to employee creativity (Unsworth et al., 2005; Shin et al., 2017). Third, we explain how group-level processes (e.g., team knowledge sharing) serve as boundary conditions through which creative process engagement affects employee creativity. Our study on the moderating role of team knowledge sharing provides another perspective for a broader understanding of the role of team knowledge sharing in promoting employee creativity, it responds to suggestions for more research into the boundary conditions under which job creativity requirement influence employee creativity (Hon, 2013; Shin et al., 2017).

## Theoretical Framework and Hypothesis

**FIGURE 1:**
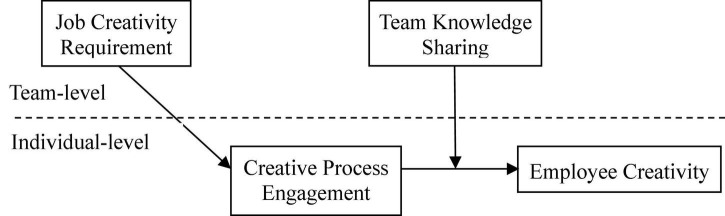
Theoretical model.

In this study, we elucidated mechanisms through which engagement in the creative process affects the creativity of employees by considering team knowledge sharing as a potential moderator. We incorporated team knowledge sharing as a moderating variable to explain how the requirement of job creativity affects the creativity of employees through creative process engagement. Figure 1 shows the hypothesized model.

### Pygmalion Theory

The Pygmalion effect is a type of a self-fulfilling prophecy that indicates how positive expectations enhance productivity and performance (Sutton and Woodman, 1989; Duan et al., 2017). Studies on management have evaluated how managers’ expectancy positively affect the performance and productivity of employees based on Pygmalion theory (Tierney and Farmer, 2011; Qu et al., 2015). The influence of the Pygmalion process depends on employees’ perception regarding organizational expectations (Eden, 1992).

We adopted Pygmalion theory (Eden, 1992) to propose that job creativity requirement can enhance employee creativity through the Pygmalion process; the level of improvement in employees’ creativity depends on how employees perceive organizational expectations toward their job roles. Accordingly, we proposed that the requirement of job creativity might more likely convey the expectations of creative outcomes to followers. This can further strengthen followers’ perception regarding their creative role and thus their engagement in the creative process. The Pygmalion process might shape creativity because the Pygmalion effect is prominent when a high level of uncertainty and risk is involved in desired performance or behaviors (Tierney and Farmer, 2004) including creativity. In addition, according to Pygmalion theory, followers are the active agents but not passive recipients in terms of organizational expectations (Karakowsky et al., 2012). Pygmalion theory can be adopted to evaluate whether followers would accept and ascribe to organizational expectations.

### Job Creativity Requirement Affects Employee Creativity Through Creative Process Engagement

Job creativity requirement is an aspect of job design that encourages components particularly included in the job description, namely autonomy, task complexity, and creativity (Unsworth et al., 2005). Employees may use new approaches or ideas to fulfill their work tasks when creativity is included as a crucial component in the job description. However, when employees might have minimal cues for their job tasks, they may use their own judgment to decide the sufficiency of work efforts. This, in turn, can result in confusion regarding the importance of creativity. Thus, employees can evaluate their progress in any job task when the requirement of job creativity is set as an organizational objective.

In creativity studies, the requirement of job creativity is viewed as a particular type of goal that should result in creative performance or output (Unsworth et al., 2005). From a sociopolitical perspective, job creativity requirement represents external demands and expectations for the creativity of employees (Yuan and Woodman, 2010). The influence of the expectations of an organization on employee behavior is dependent on employees’ interpretations of those expectations (Jiang and Gu, 2017).

Most studies on creativity have examined creativity outcomes (Amabile et al., 2005; Park et al., 2016), ignoring activities that lead to these outcomes (Gilson and Shalley, 2004). Engagement in the creative process is the necessary first step toward achieving a creative output (Zhang and Bartol, 2010; Du et al., 2016). Creative process engagement refers to the involvement of employees in methods that would yield creative output. The following three stages comprise the creative process: identifying problems, searching and encoding related information, and generating ideas (Zhang and Bartol, 2010). Here, we considered the creative process engagement of employees as a specific type of job role.

According to Sutton and Woodman (1989), the Pygmalion effect would be more prominent when performance involves more challenges and high uncertainty, as observed in creativity (Amabile et al., 1996). Considering that the Pygmalion model is crucial for explaining organizational expectations, resulting behaviors, and performance, we used this model as a conceptual framework for examining complexities related to interactions between organizations and employees and thus creative outcomes.

We hypothesized that job creativity requirement substantially affects employees’ willingness to involve in the creative process. In particular, when employees perceive their job requirement to be meaningful and personally important, they will expend more effort in understanding problems from various perspectives, thus finding a solution, using extensive information from multiple sources, and generating numerous alternative ideas by connecting different information sources (Gilson and Shalley, 2004). Furthermore, such employees are more willing to take chances, create innovative strategies, and explore different cognitive pathways (Amabile et al., 1996). On the basis of the aforementioned findings, we propose the following hypothesis:

**Hypothesis 1.** Job creativity requirement is positively associated with creative process engagement.

Engaging in a creative process differs from the general rational decision making and problem solving. A creative process can be employed to solve more ill-defined problems rather than standard problems. Furthermore, a creative process can help in objectively developing novel solutions. Moreover, the creative process involves searching, encoding, combining, or reorganizing information (Henker et al., 2015).

Identification of the problem is the first stage of the creative process (Zhang and Bartol, 2010). In this step, an employee is required to identify and structure a problem and accordingly determine goals, information, procedures, and restrictions that can be used to solve the identified problem (Reiter-Palmon and Illies, 2004). Once the problem is identified, the second step involves collecting and processing relevant information (Zhang and Bartol, 2010). In this step, people search for relevant information and concepts that can advance understanding regarding the identified problem (Mumford, 2000). The final step of the creative process involves developing concepts related to the problem and incorporating relevant information to devise ideas and alternatives (Zhang and Bartol, 2010). The gathered information can be combined and reorganized to advance the understanding and determine the applicability and implications of this new understanding, ultimately assisting in the development of novel ideas (Mumford, 2000).

A study (Zhang and Bartol, 2010) suggested that creative process participation can be beneficial for performance because individuals who exhibit a high level of creative process engagement would expend more efforts to identify problems, search for relevant information, and explore possibilities. Therefore, these individuals would more possibly develop novel solutions. Furthermore, the authors indicated that creative process engagement is positively associated with employee creativity. In a subsequent study, Harris et al. (2013) reported that creative process engagement can predict a new employee’s creativity level. Accordingly, we propose the following hypothesis:

**Hypothesis 2.** Creative process engagement is positively associated with employees’ creativity.

When Pygmalion theory is applied to a creativity context, it indicates how an organization’s creativity expectations for employees are associated with employee creativity through various mediating factors. Our emphasis on job creativity requirement advanced knowledge regarding the Pygmalion model by elucidating how organizational expectations can be translated into employee behaviors through creative process engagement. The requirement of job creativity and engagement in creative processes are positively linked to creativity, indicating that this intercorrelation may share variance to a certain extent. Thus, we proposed that creative process engagement can mediate the linkage of job creativity with employee creativity.

**Hypothesis 3.** creative process engagement mediates the linkage of job creativity requirement with employee creativity.

### Cross-Level Moderating Influence of Team Knowledge Sharing

According to the JD-R theory, an individual needs resource support from the organization to fulfill the organization’s work objectives. When employees perceive the resource support (e.g., team knowledge sharing) from the team, they will be willing to take the initiative to make behavioral changes in order to adapt their working ability to the job requirements. Team knowledge sharing refers to the sharing of task-relevant ideas, information, and suggestions with team members (Dong et al., 2017). To improve team creativity, leaders should not only motivate creative engagement among individuals but also promote intrateam communication and information exchange (Hargadon and Bechky, 2006; Presbitero et al., 2017). Taggar (2002) indicated that teams will reach a high creativity level when they consist of both creative members and effective processes that members will adopt to collectively approach and utilize knowledge available within the team.

Amabile et al. (1996) suggested that open communication for knowledge exchange within a team will influence how domain skills promote individual creativity. Therefore, team knowledge sharing will promote open communication in teams and exert a cross-level effect on the linkage of creative process engagement with employee creativity (Jiang et al., 2015). When the members of a team share knowledge, they understand different viewpoints and alternatives; this might mutually inspire the members of the team (Homan et al., 2007; Hirst et al., 2009). Information exchange provides additional valuable information to team members, and the knowledge will propel them to develop problem-solving strategies. The members of a team will utilize one another’s contributions to add-on to their resources. This will eventually assist employees in creating their individual creative strategies. Individual creativity will develop based on the knowledge of that individual, whereas sharing of knowledge in a team will help individuals more effectively use the existing knowledge to devise innovative strategies (Zhou et al., 2009; Carmeli and Paulus, 2014). When employees participate in the creative process, considering the personal knowledge is limited, creativity need knowledge resources support, lack of the knowledge sharing will be difficult to form effective creativity, by contrast, when employees in the process of creation, the team has sufficient knowledge resources to be Shared, they are more likely to effectively use knowledge to promote creativity.

When individuals substantially focus on a problem, exhibit creative process engagement, expose themselves to various ideas, and share knowledge with other team members, they will improve their dissimilar thinking, thus enhancing their creativity. Accordingly, the following hypothesis is proposed:

**Hypothesis 4.** The linkage of creative process engagement with employee creativity is moderated by team knowledge sharing. A high level of knowledge sharing can lead to a stronger relationship.

Considering that creative process engagement, job creativity requirement, and employee creativity are related, we investigated whether team knowledge sharing can moderate the influence of job creativity requirement on employees’ creativity through engagement in the creative process. Accordingly, the following hypothesis is proposed:

**Hypothesis 5.** team knowledge sharing moderates the association of job creativity requirement with employee creativity through creative process engagement. This mediated relationship would be stronger when knowledge sharing is high.

## Materials and Methods

### Sample and Procedure

This study was performed using data obtained from three IT companies located in a high-technology development area in the middle of China. The R&D teams of these companies comprised professional-level employees who worked interdependently including new product developers and software engineers. During work hours, we distributed questionnaires to all 71 supervisors of the three companies’ R&D teams and their 486 subordinates; subsequently, we collected filled questionnaires. Before distributing the questionnaires, through their human resources department, we sent a letter to participating employees and their supervisors to obtain their consent for voluntary participation and assured them regarding the confidentiality of individual responses. We allowed participants to fill the questionnaires during work hours. All supervisors completed questionnaires that assessed the creativity of their subordinates. A total of 453 subordinates completed the self-report survey (response rate = 93.21%). The average age of supervisors was 33.08 [standard deviation (SD) = 4.15] years. Furthermore, 58 (81.69%) of the 71 supervisors were men. The average organizational tenure of supervisors was 5.23 (*SD* = 2.63) years. Moreover, 297 (65.56%) of the 453 subordinates were men. The average age and organizational tenure of subordinates were 31.21 (*SD* = 4.53) and 3.47 (*SD* = 2.41) years, respectively.

Data collection was carried out in two stages, 2 months apart, in order to reduce the common method variance problem. In the first survey (Time 1), participants reported their perceptions of job creativity requirement In the second survey conducted 2 months later (Time 2), participants described their creative process engagement and team knowledge sharing, their supervisors rated participants’ creativity. We used identifiers on the questionnaire to link the two stages of the questionnaire. All the questionnaires were translated in Chinese from English by using the translation-back-translation procedure recommended by Brislin (1980). Unless indicated otherwise, we measured responses on a 5-point Likert scale; the responses ranged from 1 (strongly disagree) to 5 (strongly agree).

### Measures

#### Job Creativity Requirement

We examined job creativity requirement by employing the 5-item scale developed by Yuan and Woodman (2010). A sample scale item is as follows: “Trying new ways to solve problems is one of the requirements of my job.” Cronbach’s alpha was 0.87. In support of aggregation, the median rwg (j) across the teams was 0.93, indicating a high level of within-team agreement. Additional support for aggregating team knowledge sharing scores to the team level was provided by inter rater reliability indices [intraclass correlation (ICC) (1) = 0.46 and ICC (2) = 0.71].

#### Creative Process Engagement

We evaluated engagement in the creative process by using the scale developed by Zhang and Bartol (2010). This scale included 11 items related to three dimensions: identifying a problem, searching for information, and generating ideas. Sample items of this scale are “I spend considerable time to understand the problem,” “I search for information from multiple sources,” and “I spend considerable time in going through information that helps to develop new ideas.” Cronbach’s alpha for the three dimensions were 0.77, 0.77, and 0.81, respectively.

#### Team Knowledge Sharing

We examined team knowledge sharing by employing a 4-item scale developed by He et al. (2014). The scale’ sample item is as follows: “Our team members share ideas regarding jobs with each other.” Cronbach’s alpha was 0.93. In support of aggregation, the median rwg (j) across the teams was 0.91, indicating a high level of within-team agreement. Additional support for aggregating team knowledge sharing scores to the team level was provided by inter rater reliability indices [ICC (1) = 0.75 and ICC (2) = 0.64].

#### Employee Creativity

We evaluated employee creativity by employing a 4-item scale developed by Farmer et al. (2003). The scale’s sample item is as follows: “This employee attempts using new ideas or methods first.” Cronbach’s alpha was 0.92.

#### Control Variables

We controlled for age, team tenure, sex at the individual level, these variables can all affect the learning and creativity of individuals (Gong et al., 2013). We also control for leader-member social exchange (LMX), we do this to exclude an alternative explanation, which assumes that the creativity generated by subordinates under the requirements of innovation can be attributed to a more general positive social exchange between leaders and subordinates. LMX was assessed by employee using the seven-item scale from Bernerth et al. (2007). Furthermore, we controlled for team size at the team level; in previous studies, team size has been found to negatively affect creativity (Kratzer et al., 2008). We also controlled for team task interdependence because it has a significant impact on team knowledge sharing (Gong et al., 2013). Task interdependence was assessed by employee using the four-item scale from Kirkman et al. (2004) and aggregated to the team level. We found that the addition of these control variables did not have a significant impact on the results of the study. Therefore, as suggested by Becker (2005), we omitted LMX and task interdependence from the subsequent analysis. Finally, considering that the data was collected from three companies, we also controlled for the potential influence of companies by using company dummy (company 3 is the reference group) (Cuyper and Witte, 2006).

### Analytical Strategy

Job creativity requirement is a complex multilevel phenomenon. Most studies have operationalized the requirement of job creativity as a construct of employees’ individual perceptions. A study (Kim et al., 2010) reported that job creativity requirement can serve as an organizational objective that employees can use to monitor their progress in a task. Hence, job creativity requirement should be considered a team-level issue. We adopted cross-level strategies to determine the role of job creativity requirement in organizations.

In our data, job creativity requirement and team knowledge sharing were nested within each team. Due to the nested structure of data, firstly, multilevel Confirmatory Factor Analysis (CFA) was conducted to test the dimensionality and the discriminant validity of our multi-item measures; secondly, we applied hierarchical linear modeling (HLM) analysis to test the hypothesized model by using HLM 6.06 and Mplus 7.0. Mediation hypotheses were tested via Monte Carlo simulation procedures using the open-source software R. This method was used to accurately reflect the asymmetric nature of the sampling distribution of an indirect effect (Preacher et al., 2010). We also followed Preacher et al. (2010) in using 95% confidence intervals (CIs) to improve the statistical power to detect indirect effects in multilevel modeling.

## Results

### Preliminary Analysis

Table 1 lists the means, SDs, and correlations among variables are listed. We observed the positive association of creative process engagement with employee creativity at the individual level (*r* = 0.37, *p* < 0.01). This finding preliminary supported the hypothesized relationship. All the scales showed adequate reliability (Table 1). Before hypothesis testing, to determine whether HLM is an appropriate analytical technique for examining our nested data, we used the null (intercept only) model to evaluate whether a significant between-team variance exists in outcome variables. The null model results revealed the variance of 26% in creative process engagement and 21% in employee creativity between teams, respectively, thus indicating the appropriateness of HLM for hypothesis testing.

**TABLE 1:** Descriptive statistics, correlations, and reliabilities.

Individual-level variables	Mean	*SD*	1	2	3	4
1. Gender (1 = male 2 = female)	1.44	0.50				
2. Age	32.60	6.62	–0.17			
3. Team tenure (year)	3.03	1.81	0.26	0.27		
4. Creative process engagement	3.81	0.67	0.09	–0.06	0.06	(0.89)
5. Employee creativity	3.41	0.56	0.04	–0.07	0.06	0.37** (0.92)

**Team-level variables**	**Mean**	**SD**	**1**	**2**		

1. Team size	6.38	1.45				
2. Job creativity requirement	3.54	0.54	–0.02	(0.87)		
3. Team knowledge sharing	3.53	0.76	0.08	–0.11	(0.91)	

n = 453 for individual-level variables and n = 71 for team-level variables. Reliabilities for the scales are in parentheses and presented along the diagonal. *p < 0.05; **p < 0.01.

### Confirmatory Factor Analyses

Our data are nested within teams rather than independent, given the non-independence of nested data, it is necessary to use a multilevel CFA to investigate the suitability of the model (Chen et al., 2011). We use Mplus 7.0 with maximum likelihood estimation to conduct multi-level CFA. The multilevel confirmatory factor analysis with consistent factor structure at individual level and team level showed that the two-factor model of job creativity requirement and team knowledge sharing fit the data well, χ^2^(40, *N*_team_ = 71, *N*_individual_ = 453) = 295.16, *CFI* = 0.936, *TLI* = 0.943, *SRMR*_[between]_ = 0.032, *SRMR*_(within)_ = 0.047, *RMSEA* = 0.041. We also find that the multilevel two-factor model was superior to the one-factor model which combined job creativity requirement and team knowledge sharing into a single factor, χ^2^(41, *N*_team_ = 71, *N*_individual_ = 453) = 473.32, *CFI* = 0.874, *TLI* = 0.863, *SRMR*_[between]_ = 0.132, *SRMR*_(within)_ = 0.109, *RMSEA* = 0.106; △χ^2^(△1, *N*_team_ = 71, *N*_individual_ = 453) = 178.23, *P* < 0.001.

### Hypothesis Testing

We hypothesized that job creativity requirement shows a positively association with creative process engagement (Hypothesis 1). As presented in Model 1 (Table 2), at the between-person level, job creativity requirement showed a positive association with creative process engagement (γ = 0.35, *p* < 0.01), thus supporting Hypothesis 1.

**TABLE 2:** Hierarchical linear model (HLM) analysis results.

Variable	CPE	Employee creativity				
		
	Model1	Model2	Model3	Model4	Model5	Model6
Intercept	3.75** (0.04)	3.36** (0.03)	3.36** (0.04)	3.36** (0.03)	3.36** (0.04)	3.36** (0.03)
**Level 1 variables**						
Age	0.03 (0.01)	0.01 (0.01)	0.01 (0.01)	0.02 (0.00)	01 (0.00)	0.02 (0.00)
Gender	–0.09 (0.08)	–0.06 (0.07)	–0.05 (0.06)	–0.05 (0.06)	–0.02 (0.05)	–0.02 (0.05)
Team tenure	0.06 (0.04)	0.03 (0.03)	–0.02 (0.02)	–0.02 (0.02)	–0.02 (0.02)	–0.02 (0.02)
CPE			0.31** (0.05)	0.31** (0.06)	0.29** (0.05)	0.29** (0.05)
**Level 2 variables**						
Company 1	0.10 (0.06)	0.11 (0.06)	0.16 (0.07)	0.09 (0.05)	0.06 (0.03)	0.05 (0.03)
Company 2	0.09 (0.04)	0.10 (0.05)	0.13 (0.06)	0.08 (0.04)	0.07 (0.03)	0.04 (0.02)
Team size	0.12 (0.04)	0.05 (0.03)	0.06 (0.03)	0.04 (0.02)	0.03 (0.02)	0.04 (0.02)
JCR	0.35** (0.11)	0.34** (0.08)		0.24** (0.07)		0.17* (0.09)
TKS					0.09 (0.07)	0.08 (0.06)
**Cross-level interaction variables**						
CPE X TKS					0.26** (0.06)	0.24** (0.07)
–2log	825.16	692.37	653.19	626.23	621.04	598.11
*R* ^2^	0.08	0.10	0.13	0.28	0.29	0.33

*CPE, creative process engagement; TKS, team knowledge sharing; JCR, job creativity requirement.*

*Parenthetical values indicate standard errors. n = 453 for individual-level variables and n = 71 for team-level variables. We calculate R^2^ according to proportional change of Level 1 and Level 2 error variance because of predictors added in the models of Table 2 (Snijders and Bosker, 1999).*

*p < 0.05; **p < 0.01.

We hypothesized the presence of a positive relationship between creative process engagement and employee creativity (Hypothesis 2). As depicted in Model 3 (Table 2), at the within-person level, we observed a positive relationship between creative process engagement and employee creativity (γ = 0.31, *p* < 0.01), thus supporting Hypothesis 2.

We hypothesized that creative process engagement mediates the relationship between job creativity requirement and employee creativity (Hypothesis 3). As shown in Model 4 (Table 2), at the between-person level, job creativity requirement was positively related to creative process engagement (γ = 0.24, *p* < 0.01). Furthermore, creative process engagement showed a positive relationship with employee creativity (γ = 0.31, *p* < 0.01). We employed parametric bootstrapping to test the hypothesized cross-level of the 2–1–1 model for the indirect relationships (Preacher et al., 2010). When we used 20,000 Monte Carlo replications, we observed that job creativity requirement was a positively and indirectly related to employee creativity through creative process engagement (indirect effect = 0.127, 95% bias-corrected bootstrap CI = 0.031–0.194), thus supporting Hypothesis 3.

**FIGURE 2:**
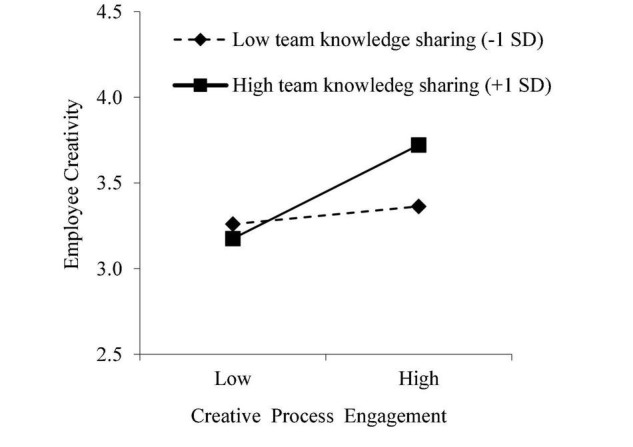
Team knowledge sharing moderates the effect of creative process engagement on employee creativity.

We proposed that a high level of team knowledge sharing results in a stronger association of creative process engagement with employee creativity (Hypothesis 4). The HLM results presented in Model 5 (Table 2) indicated that the interaction between creative process engagement and team knowledge sharing showed a positive association with employee creativity (γ = 0.26, *p* < 0.01). According to the recommendations of Cohen et al. (2003), we plotted the interaction by using the conditional values of team knowledge sharing (1 SD higher and lower than the mean). As shown in Figure 2, a higher level of team knowledge sharing (γ = 0.488, *p* < 0.01) resulted in a stronger association of creative process engagement with employee creativity (γ = 0.092, ns), thus supporting Hypothesis 4.

We proposed that team knowledge sharing moderates the linkage of job creativity requirement with employee creativity through creative process engagement (Hypothesis 5). To test this hypothesis, we investigated the indirect linkage of job creativity requirement with outcomes through engaging in the creative process at higher (+1 SD) and lower levels (–1 SD) of team knowledge sharing by using the method reported by Bauer et al. (2006). We found the indirect effect of job creativity requirement on employee creativity through creative process engagement was significantly moderated (Δγ = 0.072, *p* < 0.05). Especially, the indirect effect was stronger with high team knowledge sharing (γ = 0.136, *p* < 0.05) than low team knowledge sharing (γ = 0.064, ns), thus supporting Hypothesis 5.

## Discussion

The current study enhances our understanding regarding mechanisms through which job creativity requirement promotes employee creativity from the viewpoint of the Pygmalion process. We proposed that job creativity requirement can improve employee creativity through the Pygmalion mechanism. This field study conducted in China indicated that job creativity requirement indirectly facilitated employee creativity through creative process engagement. Furthermore, team knowledge sharing was determined to be a crucial cross-level factor mediating the indirect association of job creativity requirement with employee creativity through creative process engagement. In addition, we observed that the positive indirect linkage of job creativity requirement with employee creativity was stronger when the level of team knowledge sharing was higher.

### Theoretical Implications

This study investigated the influence of job creativity requirement, which is a vital workplace factor contributing to or affecting the creativity of employees (Shalley et al., 2004; Yuan and Woodman, 2010). Studies have reported inconsistent findings regarding the association of job creativity requirement with employee creativity (Gilson and Shalley, 2004; Unsworth et al., 2005; Yuan and Woodman, 2010; Tierney and Farmer, 2011; Shin et al., 2017). Our findings elucidate mechanisms underlying the relationship between the requirement of job creativity and the creativity of employees. Limited studies have elucidated mechanisms underlying this association. Thus, mechanisms through which the requirement of job creativity enhances employee creativity remain unclear. This study sheds light on how creative process engagement mediates the relationship between job creativity requirement and employee creativity.

Our study uses the Pygmalion effect to examine “why can you be creative if your organization requires you to be innovative?” The findings further suggest that in work environments that require innovation, when performance criteria is based on creativity. The Pygmalion framework is a relevant and actionable model for understanding employee creativity, it helps to provide guidance for employees to carry out their work creatively. The theoretical contributions of this study are described as follows.

First, this paper offers an alternative view from the perspective of the Pygmalion model to understand how job creativity requirement affects employee creativity. In contrast to previous studies examining job stress or sense-making perspective (Hon, 2013; Shin et al., 2017), we suggested that job creativity requirement can evoke the Pygmalion model to promote employee creativity through employees’ creative process engagement. We observed that the Pygmalion mechanism motivated employees to engage in the creative process. This finding indicated that when organizations expect job creativity, employees would consider creativity as their job task and thus participate in the upward creative process. Therefore, we emphasized on the Pygmalion mechanism that inherently originates from the intention of the requirement of job creativity and has been neglected in previous studies on creativity examining the importance of job creativity requirement.

Second, the results revealed that creative process engagement mediated the association of job creativity requirement with employee creativity. We explored job creativity requirement as the antecedent of creative process engagement. We built arguments and exhibited an association of job creativity requirement with creative process engagement. Previous studies have called for determining processes that individuals adopt to achieve creative outcomes (Zhang and Bartol, 2010). However, no study has determined the linkage of job creativity requirement with employee creativity. In particular, this study contributes to the creativity literature by indicating how engagement in the creative process can improve employees’ creativity. The influence of job creativity requirement on creativity partly results from its direct effect on creative process engagement. Thus, the findings support the call for additional research in the field of creative process engagement (Shalley et al., 2004; Zhang and Bartol, 2010).

Third, we used individual- and group-level processes relevant to creativity to demonstrate team knowledge sharing as a crucial factor responsible for the effects of creative process engagement on employee creativity. We determined that team knowledge sharing acts as a boundary condition when job creativity requirement more effectively facilitates employee creativity through the Pygmalion mechanism. Compared with those with a lower level of team knowledge sharing, employees with a higher level of team knowledge sharing were more willing to engage in the creative process. This allowed them to form increased creativity. Thus, although knowledge shared by other team members may promote individual creativity, individuals may benefit in different ways. In particular, knowledge sharing would be more beneficial for employees with a higher level of engagement in the creative process because knowledge exchange within a team can promote the development of employees (García-Morales et al., 2008; Dong et al., 2017). Furthermore, we observed that the indirect influence of job creativity requirement on employees’ creativity through creative process engagement was stronger when the level of team knowledge sharing was higher. These results suggest that organizations can improve employees’ creativity by enabling their participation in the creative process. Thus, team knowledge sharing can be beneficial, particularly when individuals’ engagement in the creative process is yet to reach a high level.

Our study contributes to organizational behavior research in the context of the Pygmalion effect. The Pygmalion effect can be applicable to the setting of proactive behaviors such as employee creativity. Previous works on the Pygmalion effect in management have specifically focused on determining how a leader’s performance or creativity expectations enhance employee task and creativity performance (Natanovich and Eden, 2008; Yuan and Woodman, 2010; Tierney and Farmer, 2011). Our study addresses a valuable research need from the point of view of creative processes by highlighting the influence of job creativity requirement on the creativity of employees.

### Practical Implications

Our study results can serve as a reference for human resources management. First, although creativity was previously considered as a spontaneous extra role behavior, incorporating the creativity component into employee job requirements can help organizations generate and maintain a regular and continuous creative process (Shin et al., 2017). Our results indicate that job creativity requirement can convey clear organizational expectations, thus encouraging employees to participate in the creative process and stimulate more creativity. Apart from developing a safe environment (Kark and Carmeli, 2009; Lin et al., 2017) or ascribing to employees’ self-concept (Flaherty, 2011), managers can effectively communicate their creativity expectations to their employees through the Pygmalion mechanism to encourage employee creativity. Managers generally provide the most relevant and crucial contextual cues for employees’ creativity in organizational settings (Tierney and Farmer, 2004; Jiang and Gu, 2017). Employees would more likely participate in the creative process when they perceive their managers’ creativity expectations. Thus, managers should effectively communicate their creativity expectations. Communication strategies, such as two-way feedback, can be adopted to accurately convey organization’s creativity expectations to employees.

Second, our results indicate how creative process engagement improves employee creativity. This study determined the indirect association of job creativity requirement with employee creativity through creative process engagement. Managers should promote participation in the creative process more directly. Meaningful cues should be provided to help employees make sense of creativity engagement. Furthermore, employees should be provided the time and freedom to apply creative strategies to solve problems, thus helping them build confidence in their ability to execute creative tasks (Jiang and Gu, 2017). Leaders should provide adequate time for identifying problems, fostering information search by supplying resources, and help employees develop ideas by providing analogies (Reiter-Palmon and Illies, 2004; Henker et al., 2015).

Third, we observed that team knowledge sharing moderates the influence of job creativity requirement on the creativity of employees through the Pygmalion mechanism, suggesting that team members should share knowledge. This can help them to tap into diverse opinions and skills and cognitive reservoirs (Carmeli and Paulus, 2014). Managers can facilitate team knowledge sharing. Managers who promote the sharing of knowledge and ideas would more likely have subordinates who participate in more knowledge sharing (Carmeli et al., 2013). Managers should provide adequate opportunities to team members for knowledge sharing and reward them. Knowledge sharing should be incorporated as a key component of job performance in performance evaluation systems. This can motivate employees to share information because they know that they are being evaluated.

### Limitations and Future Research Directions

This study has some limitations that should be addressed. First, the use of time-lagged data can preclude causal inferences. This limitation can preclude the research team from ruling out the presence of reverse and reciprocal causality. Thus, future field studies or laboratory experiments should use longitudinal data to determine the causal linkage and test the hypotheses.

Second, we used supervisors’ subjective evaluations of subordinates’ creativity. Thus, the ratings of the requirement of job creativity and the creativity of employees were obtained from the same source. However, we examined these two constructs at separate time points to minimize the common method variance. Additional studies should evaluate the applicability of this model when objective creativity measures are used. In addition, we examined whether creative process engagement serves as a mediator. Other variables might contribute to the association of job creativity requirement with employee creativity. The role of other mediators in the creative process should be examined in the future.

Third, data were obtained from three business organizations in China, thus potentially limiting the applicability of findings to other cultures. In particular, supervisors exert a more significant effect on employees due to power distance and paternalism that are common in Chinese culture. However, conducting a field study in different Chinese organizations can increase the external validity of the Pygmalion effect. Although the Pygmalion effect has been studied in Western cultures, its applicability in Eastern cultures remains elusive. The findings revealed that the Pygmalion effect can be generalized to Chinese culture. Therefore, how supervisors in Chinese organizations promote more creativity by setting creativity expectations and shaping creativity perceptions should be studies. Additional studies should evaluate similar models in other cultures and attempt to replicate our results.

Fourth, this study examined job requirement as the Pygmalion agent for employee creativity. Although team members can be an alternate source of expectations, their potential effect on creativity was reported (Zhou and George, 2001). Therefore, the importance of multiple components in creativity within the Pygmalion framework should be examined. Creativity is context specific, and the association of job creativity requirement with employee creativity may manifest differently based on the context (Zhou and Hoever, 2014). Therefore, future studies examining the effect of the Pygmalion process on creativity under different settings are required.

## Conclusion

This study considered the Pygmalion mechanism to examine how job creativity requirement affects employee creativity. Our findings indicated that job creativity requirement is positively associated with employee creativity through creative process engagement. Moreover, our findings suggested that team knowledge sharing can strengthen the positive linkage of creative process engagement with employee creativity. These findings shed light on the Pygmalion effect in organizational settings and offer crucial practical implications to help managers more appropriately use the Pygmalion mechanism to motivate employees’ creativity within groups.

## Data Availability Statement

The raw data supporting the conclusions of this article will be made available by the authors, without undue reservation.

## Author Contributions

YM: conception, writing—original draft, writing—reviewing, and design of study. HZ: acquisition of data and interpretation of data. YD: methodology and editing. All authors contributed to the article and approved the submitted version.

## Conflict of Interest

The authors declare that the research was conducted in the absence of any commercial or financial relationships that could be construed as a potential conflict of interest.

## Publisher’s Note

All claims expressed in this article are solely those of the authors and do not necessarily represent those of their affiliated organizations, or those of the publisher, the editors and the reviewers. Any product that may be evaluated in this article, or claim that may be made by its manufacturer, is not guaranteed or endorsed by the publisher.

## References

[B1] Amabile T. M. Barsade S. G. Mueller J. S. Staw B. M. (2005). Affect and creativity at work. *Adm. Sci. Q.* 50 367–403.

[B2] Amabile T. M. Conti R. Coon H. Lazenby J. Herron M. (1996). Assessing the work environment for creativity. *Acad. Manag. J.* 39 1154–1184. 10.2307/256995

[B3] Anderson N. Potocnik K. Zhou J. (2014). Innovation and creativity in organizations: a state-of-the-science review and prospective commentary. *J. Manag.* 40 1297–1333. 10.1177/0149206314527128

[B4] Bauer D. J. Preacher K. J. Gil K. M. (2006). Conceptualizing and testing random indirect effects and moderated mediation in multilevel models: new procedures and recommendations. *Psychol. Methods.* 11 142–163. 10.1037/1082-989X.11.2.142 16784335

[B5] Becker T. E. (2005). Potential problems in the statistical control of variables in organizational research: a qualitative analysis with recommendations. *Organ. Res. Methods* 8 274–289. 10.1186/s12913-016-1423-5 27409075 PMC4943498

[B6] Bernerth J. B. Armenakis A. A. Field H. S. Giles W. F. Walker J. H. (2007). Leader–member social exchange (LMSX): development and validation of a scale. *J. Organ. Behav.* 28 979–1003. 10.3389/fpsyg.2020.00085 32116909 PMC7031446

[B7] Brislin R. W. (1980). “Translation and content analysis of oral and written material,” in *Handbook of Cross-Cultural Psychology*, eds Triandis H. C. Berry J. W. (Boston, MA: Allyn \& Bacon), 398–444. 10.3390/healthcare6030093

[B8] Carmeli A. Gelbard R. Reiter-Palmon R. (2013). Leadership, creative problem-solving capacity, and creative performance: the importance of knowledge sharing. *Hum. Res. Manag.* 52, 95–121. 10.1002/hrm.21514

[B9] Carmeli A. Paulus P. B. (2014). CEO ideational facilitation leadership and team creativity: the mediating role of knowledge sharing. *J. Creat. Behav.* 49 53–75. 10.1002/jocb.59

[B10] Chen X. P. Liu D. Portnoy R. (2011). A multilevel investigation of motivational cultural intelligence, organizational diversity climate, and cultural sales: evidence from us real estate firms. *J. Appl. Psychol.* 97 93–106. 10.1037/a0024697 21806296

[B11] Cohen J. Cohen P. West S. Aiken L. (2003). *Applied Multiple Regression/Correlation Analysis for the Behavioral Sciences*, 3rd Edn. Mahwah, NJ: Erlbaum.

[B12] Cuyper N. D. Witte H. D. (2006). The impact of job insecurity and contract type on attitudes, well-being and behavioural reports: a psychological contract perspective. *J. Occup. Organ. Psychol.* 79 395–409. 10.1348/096317905x53660

[B13] Dong Y. Bartol K. M. Zhang Z. X. Li C. (2017). Enhancing employee creativity via individual skill development and team knowledge sharing: influences of dual-focused transformational leadership. *J. Organ. Behav.* 38 439–458. 10.1002/job.2134

[B14] Du Y. Zhang L. Chen Y. (2016). From creative process engagement to performance: bidirectional support. *Leaders. Organ. Dev. J.* 37 966–982. 10.1016/j.concog.2019.01.002 30660927 PMC6374286

[B15] Duan J. Li C. Xu Y. Wu C. (2017). Transformational leadership and employee voice behavior: a pygmalion mechanism. *J. Organ. Behav.* 38 650–670. 10.1002/job.2157

[B16] Eden D. (1992). Leadership and expectations: pygmalion effects and other self-fulfilling prophecies in organizations. *Leaders. Q.* 3 271–305. 10.1016/1048-9843(92)90018-b

[B17] Farmer S. M. Tierney P. Kung-Mcintyre K. (2003). Employee creativity in Taiwan: an application of role identity theory. *Acad. Manag. J.* 46 618–630. 10.5465/30040653 30040653

[B18] Flaherty M. A. (2011). The effects of a holistic creativity program on the self-concept and creativity of third graders. *J. Creat. Behav.* 26 165–171. 10.1002/j.2162-6057.1992.tb01173.x

[B19] García-Morales V. J. Lloréns-Montes F. J. Verdú-Jover A. J. (2008). The effects of transformational leadership on organizational performance through knowledge and innovation. *Br. J. Manag.* 19 299–319. 10.1111/j.1467-8551.2007.00547.x

[B20] Gilson L. L. Lim H. S. Luciano M. M. Choi J. N. (2013). Unpacking the cross-level effects of tenure diversity, explicit knowledge, and knowledge sharing on individual creativity. *J. Occup. Organ. Psychol.* 86 203–222. 10.1111/joop.12011

[B21] Gilson L. L. Shalley C. E. (2004). A little creativity goes a long way: an examination of team’s engagement in creative processes. *J. Manag.* 30 453–470.

[B22] Gong Y. Kim T. Y. Lee D. R. Jing Z. (2013). A multilevel model of team goal orientation, information exchange, and creativity. *Acad. Manag. J.* 56 827–851. 10.5465/amj.2011.0177

[B23] Hargadon A. B. Bechky B. A. (2006). When collections of creatives become creative collectives: a field study of problem solving at work. *Organ. Sci.* 17, 484–500. 10.1287/orsc.1060.0200 19642375

[B24] Harris T. B. Li N. Boswell W. R. Zhang X. A. Xie Z. (2013). Getting what’s new from newcomers: empowering leadership, creativity, and adjustment in the socialization context. *Pers. Psychol.* 67 567–604.

[B25] He H. Baruch Y. Lin C. P. (2014). Modeling team knowledge sharing and team flexibility: the role of within-team competition. *Hum. Relat.* 67 947–978.

[B26] Henker N. Sonnentag S. Unger D. (2015). Transformational leadership and employee creativity: the mediating role of promotion focus and creative process engagement. *J. Bus. Psychol.* 30 235–247. 10.1007/s10869-014-9348-7

[B27] Hirst G. Van Knippenberg D. Zhou J. (2009). A cross-level perspective on employee creativity: goal orientation, team learning behavior, and individual creativity. *Acad. Manag. J.* 52 280–293.

[B28] Homan A. C. Van Knippenberg D. Van Kleef G. A. De Dreu C. K. W. (2007). Bridging fault lines by valuing diversity: diversity beliefs, information elaboration, and performance in diverse work groups. *J. Appl. Psychol.* 92 1189–1199. 10.1037/0021-9010.92.5.1189 17845079

[B29] Hon A. H. Y. (2013). Does job creativity requirement improve service performance? A multilevel analysis of work stress and service environment. *Int. J. Hosp. Manag.* 35 161–170. 10.1016/j.ijhm.2013.06.003

[B30] Hughes D. J. Lee A. Tian A. W. Newman A. Legood A. (2018). Leadership, creativity, and innovation: a critical review and practical recommendations. *Leaders. Q.* 29 549–569. 10.1186/s13012-016-0452-0 27490260 PMC4977475

[B31] Jiang W. Gu Q. (2017). Leader creativity expectations motivate employee creativity: a moderated mediation examination. *Int. J. Hum. Resour. Manag.* 28 724–749.

[B32] Jiang W. Gu Q. Wang G. G. (2015). To guide or to divide: the dual-side effects of transformational leadership on team innovation. *J. Bus. Psychol.* 30 677–691.

[B33] Karakowsky L. Degama N. Mcbey K. (2012). Facilitating the Pygmalion effect: the overlooked role of subordinate perceptions of the leader. *J. Occup. Organ. Psychol.* 85 579–599. 10.1111/j.2044-8325.2012.02056.x

[B34] Kark R. Carmeli A. (2009). Alive and creating: the mediating role of vitality and aliveness in the relationship between psychological safety and creative work involvement. *J. Organ. Behav.* 30 785–804.

[B35] Kim K. Choi S. B. (2017). Influences of creative personality and working environment on the research productivity of business school faculty. *Creat. Res. J.* 29 10–20.

[B36] Kim T. Y. Hon A. H. Y. Lee D. R. (2010). Proactive personality and employee creativity: the effects of job creativity requirement and supervisor support for creativity. *Creat. Res. J.* 22 37–45.

[B37] Kirkman B. L. Rosen B. Tesluk P. E. Gibson C. B. (2004). The impact of team empowerment on virtual team performance: the moderating role of face-to-face interaction. *Acad. Manag. J.* 47 175–192. 10.2307/20159571

[B38] Kratzer J. Gemunden H. G. Lettl C. (2008). Balancing creativity and time efficiency in multi-team R&D projects: the alignment of formal and informal networks. *R D Manag.* 38 538–549. 10.1111/j.1467-9310.2008.00528.x

[B39] Kwan H. K. Zhang X. Liu J. Lee C. (2018). Workplace ostracism and employee creativity: an integrative approach incorporating pragmatic and engagement roles. *J. Appl. Psychol.* 103 1358–1366. 10.1037/apl0000320 29963897

[B40] Lin B. Law K. Zhou J. (2017). Why is underemployment related to creativity and OCB? A task crafting explanation of the curvilinear moderated relations. *Acad. Manag. J.* 60 156–177.

[B41] Mumford M. D. (2000). Managing creative people: strategies and tactics for innovation. *Hum. Resour. Manag. Rev.* 10 313–351. 10.1097/JPN.0b013e3181c94a24 20147828

[B42] Natanovich G. Eden D. (2008). Pygmalion effects among outreach supervisors and tutors: extending sex generalizability. *J. Appl. Psychol.* 93 1382–1389. 10.1037/a0012566 19025254

[B43] Park N. K. Chun M. Y. Lee J. (2016). Revisiting individual creativity assessment: triangulation in subjective and objective assessment methods. *Creat. Res. J.* 28 1–10. 10.1080/10400419.2016.1125259

[B44] Preacher K. J. Zyphur M. J. Zhang Z. (2010). A general multilevel sem framework for assessing multilevel mediation. *Psychol. Methods* 15 209–233. 10.1037/a0020141 20822249

[B45] Presbitero A. Roxas B. Chadee D. (2017). Effects of intra-and inter-team dynamics on organizational learning: role of knowledge-sharing capability. *Knowl. Manag. Res. Pract.* 15 146–154.

[B46] Qu R. Janssen O. Shi K. (2015). Transformational leadership and follower creativity: the mediating role of follower relational identification and the moderating role of leader creativity expectations. *Leaders. Q.* 26 286–299. 10.1016/j.leaqua.2014.12.004

[B47] Reiter-Palmon R. Illies J. J. (2004). Leadership and creativity: understanding leadership from a creative problem-solving perspective. *Leaders. Q.* 15 55–77.

[B48] Shalley C. E. Zhou J. Oldham G. R. (2004). The effects of personal and contextual characteristics on creativity: where should we go from here? *J. Manag.* 30 933–958.

[B49] Shin S. J. Yuan F. Zhou J. (2017). When perceived innovation job requirement increases employee innovative behavior: a sensemaking perspective. *J. Organ. Behav.* 38 68–86. 10.1002/job.2111

[B50] Snijders T. Bosker R. (1999). *An Introduction to Basic and Advanced Multilevel Modeling.* London: Sage Publications.

[B51] Sutton C. D. Woodman R. W. (1989). Pygmalion goes to work: the effects of supervisor expectations in a retail setting. *J. Appl. Psychol.* 74 943–950.

[B52] Taggar S. (2002). Individual creativity and group ability to utilize individual creative resources: a multilevel model. *Acad. Manag. J.* 45 315–330.

[B53] Tierney P. Farmer S. M. (2004). The Pygmalion process and employee creativity. *J. Manag.* 30 413–432. 10.1016/j.jm.2002.12.001

[B54] Tierney P. Farmer S. M. (2011). Creative self-efficacy development and creative performance over time. *J. Appl. Psychol.* 96 277–293. 10.1037/a0020952 20954756

[B55] Unsworth K. Wall T. Carter A. (2005). Creative requirement: a neglected construct in the study of employee creativity? *Group Organ. Manag.* 30 541–560.

[B56] Unsworth K. L. Clegg C. W. (2010). Why do employees undertake creative action? *J. Occup. Organ. Psychol.* 83 77–99.

[B57] Yuan F. Woodman R. W. (2010). Innovative behavior in the workplace: the role of performance and image outcome expectations. *Acad. Manag. J.* 53 323–342. 10.5465/amj.2010.49388995

[B58] Zhang X. Bartol K. M. (2010). Linking empowering leadership and employee creativity: the influence of psychological empowerment, intrinsic motivation, and creative process engagement. *Acad. Manag. J.* 53 107–128. 10.5465/amj.2010.48037118

[B59] Zhou J. George J. M. (2001). When job dissatisfaction leads to creativity: encouraging the expression of voice. *Acad. Manag. J.* 44 682–696.

[B60] Zhou J. Hoever I. J. (2014). Research on workplace creativity: a review and redirection. *Soc. Sci. Electron. Publ.* 1 333–359.

[B61] Zhou J. Shin S. J. Brass D. J. Choi J. Zhang Z. X. (2009). Social networks, personal values, and creativity: evidence for curvilinear and interaction effects. *J. Appl. Psychol.* 94 1544–1552. 10.1037/a0016285 19916661

